# Prevalence of voice disorders in military personnel: a systematic review and meta-analysis

**DOI:** 10.3389/fpubh.2026.1942500

**Published:** 2026-09-16

**Authors:** Joanna Morawska, Piotr Politański, Wiktoria Nowak, Kamila Przegalińska, Karolina Mędrala, Wioletta Pietruszewska

**Affiliations:** 1 Department of Otolaryngology, Head and Neck Oncology, Medical University of Lodz, Łódź, Poland; 2 Department of Electromagnetic Hazards, Nofer Institute of Occupational Medicine, Łódź, Poland; 3 Information and Library Center, Medical University of Lodz, Łódź, Poland

**Keywords:** dysphonia, inhalational exposure, military personnel, occupational exposure, occupational health, prevalence, vocal load, voice disorders

## Abstract

**Background:**

Voice and related laryngeal disorders may affect communication, occupational functioning and readiness in military populations, but their prevalence remains poorly characterized. We estimated prevalence across general military populations, high-vocal-demand personnel and chemically exposed military or veteran cohorts.

**Methods:**

This systematic review and meta-analysis followed PRISMA 2020. PubMed, Embase, Scopus, Web of Science, ClinicalTrials.gov and Google Scholar were searched in September 2024. Eligible studies included active-duty personnel, recruits, reservists or veterans and reported voice or related laryngeal outcomes. Clinically referred or symptom-selected cohorts were synthesized narratively. Subgroup-specific random-effects meta-analyses used logit-transformed proportions, restricted maximum likelihood estimation and Hartung–Knapp adjustment. Prediction intervals and generalized linear mixed-model sensitivity analyses were calculated. Risk of bias and certainty of evidence were assessed using design-appropriate appraisal tools and GRADE.

**Results:**

Sixteen studies were included in the systematic review, and 11 independent datasets from nine studies contributed to the meta-analysis. In general military populations, pooled prevalence was 3.3% (95% CI 0.2–37.5%; five datasets; *n* = 1,613,921; I^2^ = 99.8%; 95% prediction interval 0.001–99.1%). Prevalence was 40.0% in high-vocal-demand personnel (95% CI 0.8–98.2%; three datasets; *n* = 3,708; I^2^ = 99.5%; prediction interval 0–100%) and 72.8% in chemically exposed cohorts (95% CI 43.3–90.4%; three datasets; *n* = 145; I^2^ = 57.5%; prediction interval 0.6–99.9%). An exploratory meta-regression identified an overall subgroup association (*p* = 0.0300), but pairwise findings were inconsistent and statistical power was limited. Certainty of evidence was Very Low for all three estimates.

**Conclusions:**

Reported prevalence varies substantially across military populations and exposure contexts. Because heterogeneity was high and the certainty of evidence was Very Low, the pooled estimates should be interpreted with caution and should not be considered precise prevalence figures or evidence of causation. Standardized prospective studies are needed to quantify the occupational-health burden and evaluate targeted surveillance, prevention and early-referral approaches.

**Systematic review registration:**

PROSPERO https://www.crd.york.ac.uk/PROSPERO/view/CRD420250640753, identifier: CRD420250640753.

## 1 Introduction

Effective spoken communication is central to military work. Military leadership doctrine emphasizes communication, command and coordination as core elements of effective military functioning (1). Commands, training instructions and operational coordination often need to be delivered in noisy, physically demanding and time-critical environments. In such settings, the voice is not only a biological function but also an occupational tool. Impaired voice production may therefore affect communication reliability, training performance, leadership, safety and operational readiness (2–5).

Military personnel may be exposed to several work-related risk factors for voice disorders. High vocal load is common in roles requiring sustained or forceful voice use, such as drill instruction, command, physical training and communication in high-noise environments (2–7). Ambient noise from weapons, vehicles, aircraft and machinery can increase vocal effort and may contribute to vocal fatigue, phonotrauma and persistent dysphonia (6–11). In addition, military training and deployment may involve exposure to airborne irritants, dust, smoke and chemical agents, which can affect the larynx and upper airway (3, 12–16). Neurological injury, including traumatic brain injury, may further impair communication and vocal control (17). Combat-related respiratory and laryngeal disorders may also affect upper-airway function and voice production (18).

Although occupational voice disorders have been studied extensively in civilian voice-demanding professions, particularly teaching, their burden in military populations remains poorly quantified (6, 7, 19). Military personnel differ from civilian workers because of selection effects, fitness requirements, deployment-related exposures and the potential underreporting of symptoms that may be perceived as minor or non-operational. The “healthy warrior effect” may also lead to lower apparent morbidity in active-duty cohorts, while veteran and exposure-specific cohorts may capture longer-term consequences of service-related hazards (20, 21).

From a public-health and workforce-health perspective, the relevance of these disorders extends beyond individual symptoms. In military populations, communication capacity may be essential to instruction, coordination, safety, and operational readiness. However, the extent to which voice and related laryngeal outcomes are captured by existing occupational-health surveillance remains unclear, and the military roles or exposure contexts that may warrant targeted prevention and early referral have not been systematically identified. Synthesizing the available evidence by occupational and environmental risk group is therefore important for informing surveillance priorities and identifying gaps in preventive military health practice.

Reported prevalence estimates vary widely across military populations, ranging from low rates in broad military cohorts to substantially higher rates in personnel with intensive vocal demands or documented chemical exposure (2–5, 14–17, 22, 23). Differences in study populations, case definitions, and assessment methods have limited a clear understanding of the occupational burden of voice disorders in military settings. Because the military literature uses overlapping terms and different assessment methods, “voice and related laryngeal disorders” was used in this review as an umbrella term for the synthesis of functionally relevant outcomes rather than as a single clinical diagnosis.

To our knowledge, no systematic review and meta-analysis has synthesized the prevalence of voice and related laryngeal disorders in military personnel and veterans. The primary aim of this review was to estimate prevalence separately in general military populations, high-vocal-demand personnel and chemically exposed cohorts. Secondary aims were to explore differences between these subgroups, examine the influence of outcome definition and case ascertainment, and synthesize findings from clinically referred or symptom-selected military populations. The review was intended to characterize the available evidence and identify priorities for future occupational-health research rather than to establish causal effects or the effectiveness of screening or preventive interventions.

## 2 Methods

### 2.1 Protocol and registration

This systematic review and meta-analysis was conducted in accordance with the PRISMA 2020 statement (24). The review was registered in PROSPERO on 19 March 2025 (CRD420250640753; https://www.crd.york.ac.uk/PROSPERO/view/CRD420250640753). Registration occurred after completion of the formal literature searches and study screening but before data extraction, risk-of-bias assessment and quantitative synthesis.

The primary review question, population, outcome domain and core eligibility criteria were established before the literature search. Nevertheless, because registration occurred after study identification and screening, it did not provide fully prospective documentation of these stages. The timing of registration and its implications for methodological transparency are acknowledged as a limitation of the review. No separate full protocol document was prepared.

The final synthesis differed from the registered analysis plan in several respects because of the limited number and substantial clinical diversity of the available studies. The registered plan anticipated separate analyses according to diagnostic and risk categories, including vocal cord dysfunction, dysphonia, vocal strain and traumatic brain injury. However, the available data were insufficient for reliable diagnosis-specific pooling, and several studies reported overlapping voice and laryngeal outcomes. The quantitative synthesis was therefore organized according to occupational and environmental risk context: general military populations, high-vocal-demand personnel and chemically exposed groups. Planned trim-and-fill and Bayesian analyses were not performed because the small number of studies within each subgroup would not have supported reliable interpretation. These changes did not alter the primary review question or the core population and outcome eligibility criteria.

Two reviewers independently conducted study screening, data extraction and risk-of-bias assessment. Disagreements were resolved by discussion; if necessary, a third reviewer was consulted.

### 2.2 Eligibility criteria

Eligible studies included active-duty personnel, reservists, recruits, veterans or other former members of the armed forces. Observational study designs, including cross-sectional studies, cohort studies and retrospective database analyses, were eligible. Randomized trials were also eligible if they reported relevant baseline prevalence data, although no eligible randomized trials were identified.

The review considered voice and related laryngeal disorders as a broad outcome domain rather than as a single clinically homogeneous condition. Eligible outcomes included dysphonia, persistent hoarseness, vocal fatigue, self-reported voice impairment, vocal fold pathology, paradoxical vocal fold motion or vocal cord dysfunction, and exercise-induced laryngeal obstruction. Because these conditions differ in their clinical presentation and in how they were identified or measured, the original outcome definitions used by the individual studies were retained during data extraction and interpretation.

Differences between administrative or ICD-based diagnoses, self-reported symptoms, screening questionnaires and clinical or endoscopic assessments were examined in sensitivity analyses where the available data permitted.

For inclusion in the quantitative synthesis, studies were required to provide an identifiable sampling frame and extractable numbers of affected individuals and total participants. Studies based entirely on participants selected because of an established voice or laryngeal disorder, related symptoms or referral for specialist assessment were retained in the systematic review but were not included in prevalence pooling because their denominators did not represent an unselected military source population. These studies were synthesized narratively.

Studies were excluded if they were conducted exclusively in civilian populations, did not report original quantitative data, lacked a sufficiently defined voice or laryngeal outcome, or did not provide data relevant to the review objectives. Reports published only in languages other than English were also excluded. This language restriction was established at the review planning stage because resources for reliable translation and independent eligibility assessment, methodological appraisal and data extraction from non-English full texts were unavailable.

The cohort reported by Forbes et al. consisted of post-deployment personnel evaluated for persistent dyspnoea but was not selected specifically on the basis of a pre-existing voice or laryngeal diagnosis. It was therefore retained in the primary general military analysis. Because recruitment based on respiratory symptoms could nevertheless have increased the probability of detecting a laryngeal disorder, an additional sensitivity analysis was performed after excluding this study.

### 2.3 Search strategy and study selection

We searched PubMed and Embase on 6 September 2024, Scopus on 11 September 2024, Web of Science on 12 September 2024, ClinicalTrials.gov on 13 September 2024, and Google Scholar on 19 September 2024. Reference lists of eligible articles and relevant reviews were also hand-searched. Randomized trials were eligible if they reported relevant baseline prevalence data, but no eligible randomized trials were identified. Complete database-specific search strategies, exact search dates, applied filters, and numbers of records retrieved are provided in Supplementary File 1.

Records were managed in EndNote. After deduplication, two reviewers independently screened titles and abstracts, followed by independent full-text assessment of potentially eligible articles. Reasons for full-text exclusion were recorded. No formal statistic of inter-reviewer agreement, such as Cohen's kappa, was calculated. Disagreements were resolved through discussion and, when necessary, consultation with a third reviewer. The study-selection process is shown in the PRISMA flow diagram (Figure 1).

**Figure 1:**
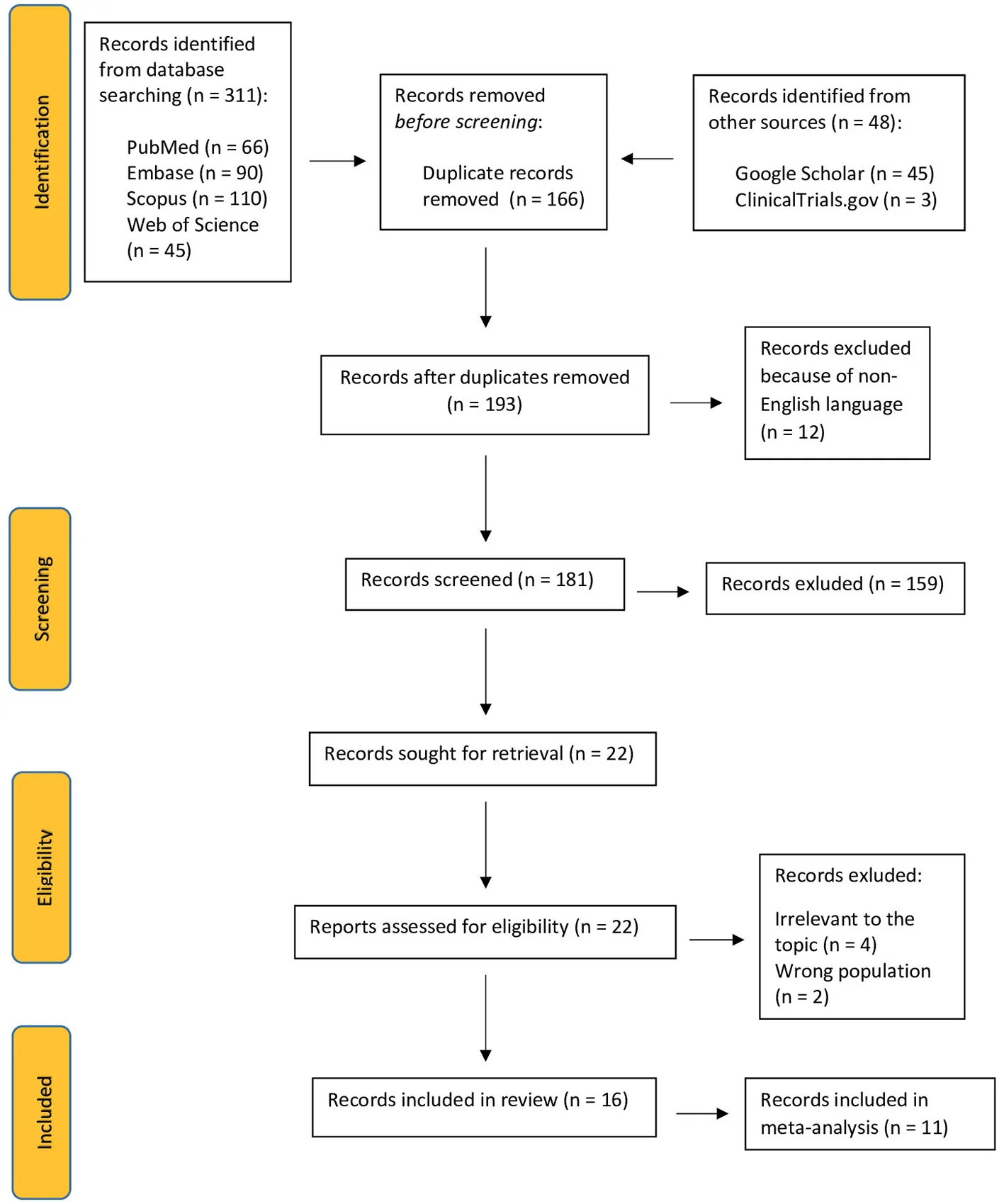
PRISMA 2020 flow diagram of study selection. Flow diagram showing record identification, screening, full-text eligibility assessment, and inclusion of studies in the systematic review and meta-analysis. PRISMA, Preferred Reporting Items for Systematic Reviews and Meta-Analyses.

### 2.4 Data extraction

Two reviewers independently extracted study characteristics, including author, year, design, country or military setting, population type, active-duty or veteran status, sample size, number of voice-disorder cases, prevalence estimate, diagnostic or screening method, occupational or environmental risk factors, and eligibility for quantitative synthesis. Extracted exposure categories included high vocal demand, chemical or inhalational exposure and traumatic brain injury where reported. Disagreements were resolved by discussion; if necessary, a third reviewer was consulted.

### 2.5 Risk of bias and certainty of evidence

Risk of bias was assessed independently by two reviewers using tools selected according to study design. Cohort studies were evaluated using the Newcastle–Ottawa Scale (25), which assesses selection, comparability and outcome ascertainment; scores of 7–9, 5–6 and 4 or fewer were interpreted as low, moderate and high risk of bias, respectively. Cross-sectional and prevalence studies were evaluated using the Joanna Briggs Institute critical appraisal checklist for studies reporting prevalence data (26), and retrospective case series were evaluated using the JBI checklist for case series (27). JBI items were rated as Yes, No, Unclear or Not applicable, and overall judgements were based on the number and seriousness of identified limitations rather than on an unvalidated numerical summary score. The certainty of evidence for the three subgroup-specific pooled prevalence estimates—general military populations, high-vocal-demand personnel and chemically exposed cohorts—was assessed using the GRADE framework (28). Two reviewers independently evaluated risk of bias, inconsistency, indirectness, imprecision and publication bias. Imprecision was judged serious when the width of the 95% confidence interval exceeded 10 percentage points and very serious when it exceeded 20 percentage points or encompassed substantially different prevalence levels. Because the evidence consisted of observational prevalence studies, certainty began at Low and was downgraded for serious or very serious concerns in the relevant domains. Publication bias could not be assessed reliably because each subgroup contained fewer than 10 studies. Disagreements were resolved through discussion or, when necessary, consultation with a third reviewer. Study-level risk-of-bias assessments and GRADE evidence profiles are provided in Supplementary Tables S2–S5.

### 2.6 Outcomes and subgroup definitions

The primary outcome was the prevalence proportion of voice or related laryngeal disorders, calculated as the number of affected individuals divided by the corresponding study population. The outcome definition reported by each study was retained. Accordingly, administrative or ICD-based diagnoses, self-reported symptoms, screening-questionnaire results and clinically assessed disorders were not treated as clinically identical outcomes. Differences according to the method of outcome identification were examined in sensitivity analyses where sufficient data were available.

Because the included military populations differed substantially in occupational demands, environmental exposures and expected baseline risk, no overall pooled prevalence was calculated across all datasets. Quantitative analyses were instead conducted within three occupationally and environmentally defined subgroups:

- General military populations: broad active-duty, recruit or veteran cohorts that were not selected on the basis of high occupational vocal demand, documented chemical exposure or a pre-existing voice or laryngeal disorder.
- High-vocal-demand personnel: military personnel in roles involving sustained, intensive or forceful voice use, including drill instructors, trainers and comparable instructional personnel.
- Chemically exposed populations: military personnel or veterans with documented exposure to chemical or inhalational irritants potentially affecting the larynx or upper airway.

Active-duty personnel, recruits and veterans were not combined in a single analysis across all exposure contexts. All of these service-status groups were eligible because military service may involve occupational and environmental hazards relevant to upper-airway health (29). They were assigned to subgroups according to the occupational or environmental context represented by each dataset. Service status was not used as a separate primary subgroup because too few datasets were available to support reliable stratified pooling. Nevertheless, because veterans may differ from active-duty personnel in exposure history, healthcare access and baseline risk, an additional sensitivity analysis was conducted after excluding the veteran dataset from the general military subgroup.

When a study reported results for two non-overlapping military populations with different occupational exposure profiles, each population was treated as an independent dataset and assigned to the appropriate subgroup. This applied to the general and high-vocal-demand populations reported by Sapir et al. and Perry et al.

### 2.7 Statistical analysis

For each eligible dataset, prevalence was calculated as the number of cases divided by the total study population. Study-specific proportions were transformed to the logit scale before pooling. Because the included populations differed substantially in occupational and environmental risk profile, no overall pooled estimate was calculated across all studies. Separate random-effects meta-analyses were performed for the general military, high-vocal-demand and chemically exposed subgroups.

Between-study variance (τ^2^) was estimated using restricted maximum likelihood (REML). Confidence intervals for the pooled estimates were calculated using the Hartung–Knapp adjustment with an *ad hoc* safeguard applied when the Hartung–Knapp standard error was smaller than the conventional random-effects standard error (30, 31). Hartung–Knapp–based 95% prediction intervals using a t-distribution with k−2 degrees of freedom were calculated to describe the expected range of prevalence in a comparable future population (32). Pooled estimates and prediction intervals were back-transformed and reported as percentages. Statistical heterogeneity was quantified using Cochran's Q, τ^2^ and I^2^ (33).

All datasets eligible for quantitative synthesis contained at least one case and at least one participant without the outcome; therefore, no continuity correction was required and no dataset was excluded from pooling solely because of a prevalence of 0% or 100%. Studies reporting 100% prevalence were retained in the systematic review but excluded from quantitative synthesis because they comprised outcome-selected symptomatic or clinically referred cohorts and were therefore unsuitable for estimating population prevalence. As a sensitivity analysis, subgroup estimates were also calculated using random-intercept generalized linear mixed models with a binomial likelihood and logit link, which can accommodate boundary proportions without continuity correction (34).

Differences between the three predefined subgroups were examined using a mixed-effects meta-regression with REML estimation and Knapp–Hartung inference. Subgroup membership was entered as a categorical moderator, and pairwise comparisons were obtained by changing the reference category (30, 35). Because only 11 datasets were available and some subgroups contained three datasets, the meta-regression was considered exploratory and its results were interpreted in conjunction with confidence intervals and residual heterogeneity.

Robustness was further examined using leave-one-out analyses within each subgroup. Additional sensitivity analyses compared studies according to outcome ascertainment, including administrative or ICD-based diagnoses, self-reported symptoms or questionnaires, and clinical or endoscopic assessment. We also repeated the general-military analysis after excluding the respiratory-symptom referral cohort reported by Forbes et al. and, separately, after excluding the veteran cohort reported by Norman et al.

Formal tests of funnel-plot asymmetry were not performed because each subgroup contained fewer than 10 datasets, making such tests underpowered and potentially misleading. Publication bias could therefore not be assessed reliably.

All revised analyses were conducted in R version 4.6.0 using the meta package version 8.5-0 and metafor package version 5.0-1. Full analytical details and sensitivity analyses are provided in Supplementary File 2.

## 3 Results

### 3.1 Study selection and study characteristics

Sixteen studies met the eligibility criteria and were included in the systematic review. The quantitative synthesis included 11 independent datasets from nine studies, comprising 1,617,774 military-affiliated participants. Seven studies were retained for narrative synthesis and were not included in the prevalence meta-analysis. Six examined clinically referred or symptom-selected populations whose denominators did not represent unselected military source populations. The remaining study was a reanalysis of a cohort already represented in the quantitative synthesis and was not pooled separately to avoid double-counting participants. The study-selection process is presented in Figure 1.

The 11 datasets included in the quantitative synthesis were classified according to occupational or environmental context. The general military subgroup comprised five datasets from broad military, recruit or veteran populations that were not selected for high occupational vocal demand or chemical exposure (2, 5, 17, 22, 23). The high-vocal-demand subgroup comprised three datasets involving drill instructors, trainers or other personnel in roles requiring sustained or forceful voice use (2, 4, 5). The chemically exposed subgroup comprised three datasets from personnel or veterans with documented exposure to chemical or inhalational irritants, including sulfur mustard, tear gas or pepper spray (14–16).

Two studies contributed more than one non-overlapping dataset. Perry et al. reported separate results for general military personnel and personnel classified as having high occupational vocal demand (2), whereas Sapir et al. reported separate results for female army recruits and instructors (5). These populations were treated as independent analytical units and assigned to the appropriate subgroups. A subsequent analysis of the Sapir cohort was not included in the quantitative synthesis to avoid double-counting participants (36).

Definitions of voice and related laryngeal outcomes varied considerably across the included studies. Studies of broad military populations included administrative or ICD-based diagnoses, self-reported symptoms and clinical assessments. High-vocal-demand studies primarily used self-reported symptoms or questionnaire-based assessments, whereas chemically exposed studies used clinical evaluation or screening for persistent laryngeal symptoms. These outcomes were retained according to the definitions used in the original studies and were not interpreted as representing a single clinically homogeneous disorder.

Traumatic brain injury was identified in the registered plan as a potential risk-based subgroup. However, no included study provided extractable prevalence data specifically for voice disorders among personnel with traumatic brain injury. TBI-related findings were therefore considered narratively and were not subjected to separate quantitative pooling. Study characteristics and eligibility for quantitative synthesis are summarized in Table 1, with additional details provided in Supplementary Table S1.

**Table 1:** Characteristics of studies and datasets included in the systematic review.

Study	Population	Sample size	Analysis
Akhavan et al. (14)	Chemical exposure	50	Meta-analysis
Balali-Mood et al. (15)	Chemical exposure	43	Meta-analysis
Dion et al. (23)	General military	1,309,015	Meta-analysis
Forbes et al. (22)	General military	108	Meta-analysis
Norman et al. (17)	General military	303,716	Meta-analysis
Perry et al. (2)	General military	696	Meta-analysis
	High vocal demand	3,427	Meta-analysis
Sapir et al. (5)	High vocal demand	130	Meta-analysis
	General military	386	Meta-analysis
Shah et al. (16)	Chemical exposure	52	Meta-analysis
Spellman et al. (4)	High vocal demand	151	Meta-analysis
Duvall and Dion (3)	Symptomatic/referred	12	Narrative
Gurevich-Uvena et al. (37)	Symptomatic/referred	265	Narrative
Morris et al. (38)	Symptomatic/referred	72	Narrative
Nolt et al. (39)	Symptomatic/referred	100	Narrative
Sapir et al. (36)	Symptomatic/reanalysis	443	Narrative
Morris et al. (40)	Symptomatic/referred	105	Narrative
Morris et al. (18)	Symptomatic/referred	48	Narrative

### 3.2 Risk of bias and certainty of evidence

Risk of bias was assessed for all 16 included studies using tools selected according to study design. Four cohort studies were evaluated using the Newcastle–Ottawa Scale and received scores of 5–6, corresponding to moderate risk of bias. Of the nine cross-sectional or prevalence studies assessed using the JBI checklist for prevalence studies, eight were rated at moderate risk and one at high risk. The principal concerns were non-random or selected sampling, inadequate sample size, limited control for potential confounding and differences in the methods used to identify or measure voice and laryngeal outcomes. Three retrospective case series were assessed using the JBI checklist for case series; one was rated at low risk and two at moderate risk. Overall, one study was rated at low risk of bias, 14 at moderate risk and one at high risk. No study was excluded solely because of its risk-of-bias assessment.

The certainty of evidence was rated as Very Low for all three subgroup-specific pooled prevalence estimates. Observational prevalence evidence initially started at Low certainty and was downgraded for serious or very serious concerns relating to risk of bias, inconsistency and imprecision. The very wide confidence and prediction intervals, substantial unexplained heterogeneity and small number of studies in the exposure-defined subgroups were the principal reasons for downgrading. Publication bias could not be assessed reliably because each subgroup contained fewer than 10 datasets. Study-level risk-of-bias assessments and the complete GRADE evidence profiles are provided in Supplementary Tables S2–S5.

### 3.3 Subgroup-specific prevalence of voice disorders

Subgroup-specific prevalence estimates were calculated using random-effects models with restricted maximum likelihood estimation and Hartung–Knapp adjustment (Table 2; Figure 2). No overall prevalence was calculated across the three occupational and environmental subgroups.

**Table 2:** Subgroup-specific pooled prevalence, prediction intervals and heterogeneity.

Subgroup	No. of datasets	Participants	Cases	Pooled prevalence, (%)(95% CI)	95% prediction interval, %	τ^2^	I^2^, %	Q (df), *p*-value
General military	5	1,613,921	6,139	3.30 (0.19–37.54)	0.001–99.06	5.3114	99.8	2,292.46 (4), *p* < 0.0001
High vocal demand	3	3,708	483	40.00 (0.80–98.22)	0–100	3.1315	99.5	389.77 (2), *p* < 0.0001
Chemical exposure	3	145	105	72.81 (43.30–90.38)	0.64–99.91	0.1448	57.5	4.70 (2), *p* = 0.0952

CI, confidence interval; PI, prediction interval; Q, Cochran's Q statistic; τ^2^, estimated between-study variance on the logit scale; I^2^, proportion of variability attributable to between-study heterogeneity. Pooled estimates were calculated using random-effects models of logit-transformed proportions with restricted maximum likelihood estimation and Hartung–Knapp adjustment. No overall estimate was calculated across the three subgroups.

**Figure 2:**
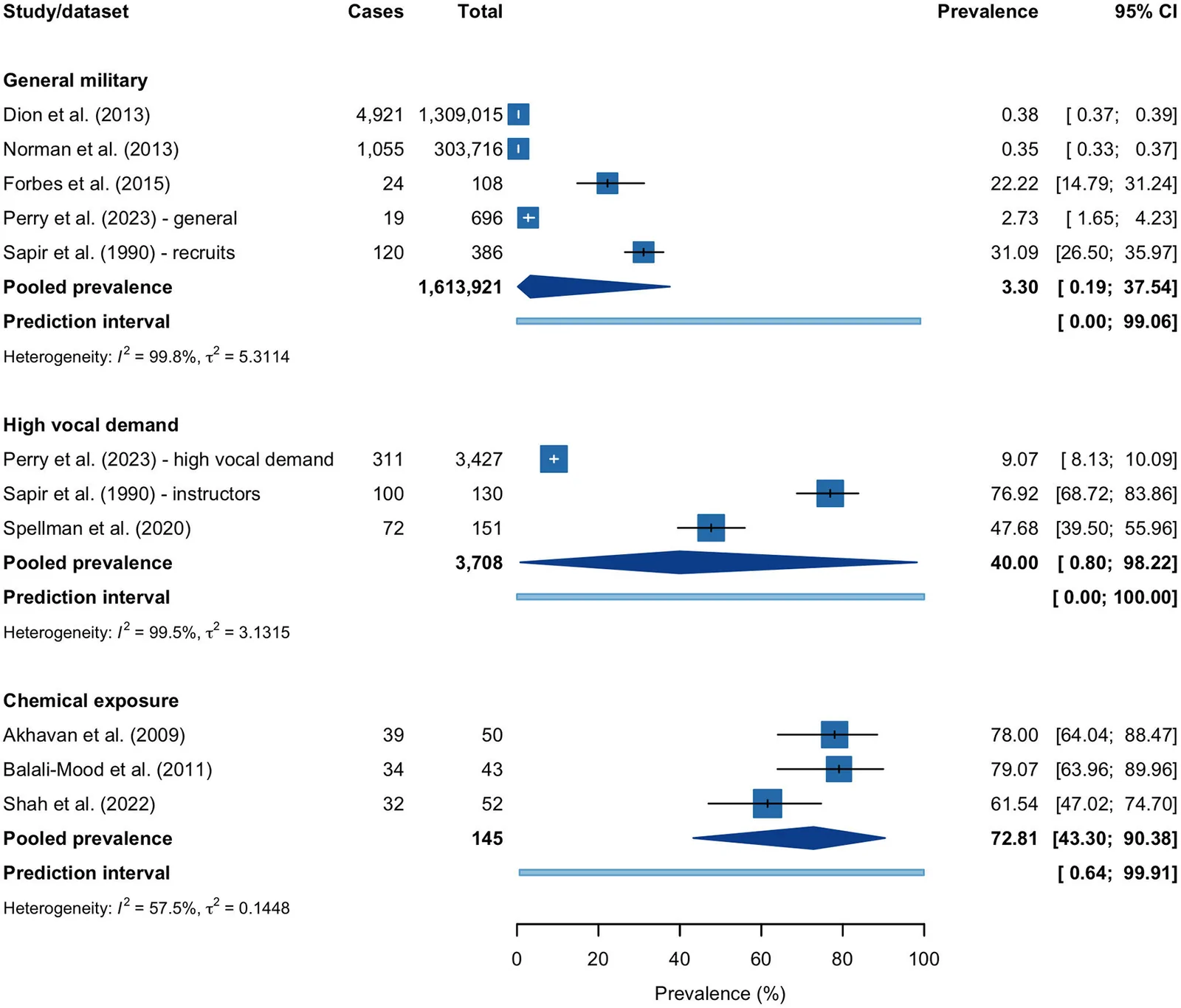
Forest plots of prevalence estimates by military subgroup. Squares represent study-specific prevalence estimates, and horizontal lines represent their 95% confidence intervals. Diamonds represent subgroup-specific pooled estimates from random-effects models using restricted maximum likelihood estimation with Hartung–Knapp adjustment. Light-blue bars represent 95% prediction intervals. No overall estimate was calculated across the three subgroups. CI, confidence interval.

The general military subgroup comprised five datasets with 6,139 cases among 1,613,921 participants. The pooled prevalence was 3.3% (95% CI 0.2–37.5%), with substantial heterogeneity (I^2^ = 99.8%; τ^2^ = 5.3114). The 95% prediction interval ranged from 0.001% to 99.1%. Study-specific prevalence estimates ranged from 0.35% in Norman et al. (17) to 31.1% among the recruits reported by Sapir et al. (5).

The high-vocal-demand subgroup comprised three datasets with 483 cases among 3,708 participants. The pooled prevalence was 40.0% (95% CI 0.8–98.2%), with substantial heterogeneity (I^2^ = 99.5%; τ^2^ = 3.1315). The 95% prediction interval ranged from approximately 0% to 100%. Study-specific prevalence estimates ranged from 9.1% in the high-vocal-demand population reported by Perry et al. (2) to 76.9% among the army instructors reported by Sapir et al. (5).

The chemically exposed subgroup comprised three datasets with 105 cases among 145 participants. The pooled prevalence was 72.8% (95% CI 43.3–90.4%), with moderate heterogeneity (I^2^ = 57.5%; τ^2^ = 0.1448). The 95% prediction interval ranged from 0.6% to 99.9%. Study-specific prevalence estimates ranged from 61.5% in Shah et al. (16) to 79.1% in Balali-Mood et al. (15).

Although the subgroup point estimates differed markedly, their confidence intervals and prediction intervals were wide and overlapped substantially. The pooled values therefore summarize the center of highly variable study-specific estimates and should not be interpreted as prevalence values expected to apply uniformly across military settings.

### 3.4 Subgroup comparisons

An exploratory mixed-effects meta-regression was conducted across the 11 datasets using subgroup membership as a categorical moderator. The omnibus test of the subgroup moderator was statistically significant [F(2, 8) = 5.61, *p* = 0.0300], indicating an overall association between subgroup membership and logit-transformed prevalence. Subgroup membership accounted for an estimated 48.1% of the between-study heterogeneity (R^2^ = 48.1%); however, residual heterogeneity remained extremely high [residual I^2^ = 99.9%; Q?(8) = 2,686.94, *p* < 0.0001].

In pairwise comparisons, the estimated logit prevalence was higher in the high-vocal-demand subgroup than in the general military subgroup, but the difference did not reach statistical significance after the small-sample adjustment (logit difference 2.9751, 95% CI −0.1923 to 6.1426; *p* = 0.0622). The chemically exposed subgroup had a higher estimated logit prevalence than the general military subgroup (logit difference 4.3987, 95% CI 1.2066 to 7.5908; *p* = 0.0130). The difference between the chemically exposed and high-vocal-demand subgroups was not statistically significant (logit difference 1.4236, 95% CI −2.1399 to 4.9870; *p* = 0.3839). Pairwise comparisons are presented in Table 3.

**Table 3:** Exploratory pairwise meta-regression comparisons between military subgroups.

Comparison	Logit difference	SE	95% CI	*p*-value
High vocal demand vs. general military	2.9751	1.3736	−0.1923 to 6.1426	0.0622
Chemical exposure vs. general military	4.3987	1.3842	1.2066 to 7.5908	0.0130
Chemical exposure vs. high vocal demand	1.4236	1.5453	−2.1399 to 4.9870	0.3839

CI, confidence interval; SE, standard error. Positive logit differences indicate a higher estimated prevalence in the first-named subgroup. The mixed-effects meta-regression included 11 datasets and used restricted maximum likelihood estimation with a small-sample adjustment. The omnibus test of subgroup membership was F(2, 8) = 5.61, *p* = 0.0300. Subgroup membership accounted for an estimated 48.1% of between-study heterogeneity, although residual heterogeneity remained extremely high (residual I^2^ = 99.9%). Results should be interpreted as exploratory because of the small number of datasets.

These results should be considered exploratory because the meta-regression included only 11 datasets, with three datasets in each of the two exposure-defined subgroups. Statistical power was therefore limited, and the study-level comparisons may also have been affected by differences in population selection and outcome definition.

### 3.5 Sensitivity analyses

The generalized linear mixed model sensitivity analyses produced pooled point estimates similar to those obtained using the primary REML–Hartung–Knapp models. The GLMM estimate was 3.3% (95% CI 0.6–17.0%) for the general military subgroup, 40.0% (95% CI 11.4–77.6%) for the high-vocal-demand subgroup and 72.9% (95% CI 62.6–81.2%) for the chemically exposed subgroup. The similarity of the point estimates indicated that the central estimates were not materially affected by the choice between the two statistical models, although the widths of their confidence intervals differed.

Sensitivity analyses according to outcome definition demonstrated substantial differences in prevalence between methods of outcome identification. Within the general military subgroup, the two datasets using ICD or administrative diagnoses produced a pooled prevalence of 0.36% (95% CI 0.22–0.60%; I^2^ = 81.6%). The two datasets based on self-reported symptoms produced a pooled prevalence of 10.2% (95% CI approximately 0–100%; I^2^ = 99.1%). The remaining clinically assessed dataset, Forbes et al., reported a prevalence of 22.2% (95% binomial CI 14.8–31.2%). All three high-vocal-demand datasets used self-reported symptoms or questionnaire-based assessments and therefore corresponded to the primary estimate of 40.0% (95% CI 0.8–98.2%; I^2^ = 99.5%).

Within the chemically exposed subgroup, the two studies using clinical dysphonia assessment produced a pooled prevalence of 78.5% (95% CI 12.9–98.9%; I^2^ = 0%). The remaining study, which used the Laryngeal Hypersensitivity Questionnaire, reported a prevalence of 61.5% (95% binomial CI 47.0–74.7%). Because the outcome-definition categories contained only one or two studies, these analyses were descriptive and should not be interpreted as providing stable alternative pooled estimates.

Exclusion of Forbes et al., whose participants had been recruited because of post-deployment dyspnoea, reduced the pooled prevalence in the general military subgroup from 3.3% to 2.0% (95% CI 0.05–43.5%; 95% prediction interval approximately 0–99.9%; I^2^ = 99.8%). Exclusion of the veteran dataset reported by Norman et al. increased the pooled prevalence to 5.7% (95% CI 0.2–67.6%; 95% prediction interval approximately 0–100%; I^2^ = 99.9%). Neither exclusion resolved the substantial heterogeneity or imprecision.

In leave-one-out analyses, pooled prevalence estimates ranged from 1.75% to 5.71% in the general military subgroup, from 23.1% to 63.4% in the high-vocal-demand subgroup and from 70.0% to 78.5% in the chemically exposed subgroup. The high-vocal-demand results were particularly sensitive to the exclusion of individual datasets, whereas the chemically exposed point estimates were comparatively stable. Confidence intervals remained wide, especially in analyses containing only two studies. Complete leave-one-out results are provided in Supplementary Table S6 and Supplementary Figure S1. The model-comparison, outcome-definition and study-exclusion sensitivity analyses are presented in Supplementary Table S7.

Formal tests of funnel-plot asymmetry and small-study effects were not performed because each subgroup contained fewer than 10 datasets. Publication bias could therefore not be assessed reliably.

### 3.6 Symptomatic and clinically referred cohorts

Seven studies were synthesized narratively and were not included in the prevalence meta-analysis. Six studies examined clinically referred or symptom-selected military populations (3, 18, 37–40). Because inclusion in these studies was conditional on the presence of voice, respiratory or upper-airway symptoms, their denominators did not represent unselected military source populations and could not provide population-based prevalence estimates. The remaining study, Sapir et al. (36), was a reanalysis of a cohort already represented by Sapir et al. (5) and was not pooled separately to avoid double-counting participants.

The clinically selected studies reported phonotraumatic vocal-fold lesions, dysphonia, paradoxical vocal fold motion or vocal cord dysfunction, chronic laryngeal inflammation and related upper-airway abnormalities among service members referred for voice or respiratory assessment. Several reported a prevalence of 100% within the selected sample. Their exclusion from quantitative synthesis was based on the sampling frame rather than on the statistical difficulty of analyzing boundary proportions; consequently, the use of continuity corrections or a generalized linear mixed model would not have made these studies suitable for estimating population prevalence. These findings nevertheless indicate a substantial clinical burden among symptomatic or referred military personnel and are summarized narratively in Table 1 and Supplementary Table S1.

## 4 Discussion

To our knowledge, this is the first systematic review and meta-analysis to synthesize the prevalence of voice and related laryngeal disorders in military-affiliated populations. The subgroup-specific pooled estimates were 3.3% in general military populations, 40.0% in high-vocal-demand personnel and 72.8% in chemically exposed cohorts. However, these estimates were accompanied by substantial or extreme heterogeneity, very wide confidence and prediction intervals, and Very Low certainty of evidence. They should therefore be interpreted as descriptive summaries of a heterogeneous evidence base rather than as precise prevalence benchmarks or evidence of a causal exposure–response relationship.

The higher point estimate observed in high-vocal-demand personnel is occupationally plausible. Military instructors, trainers and other communication-intensive personnel may be required to deliver repeated commands, speak for prolonged periods, shout and communicate in noisy environments. These conditions are consistent with recognized mechanisms of occupational voice loading and phonotraumatic risk (6, 7). Nevertheless, this subgroup contained only three datasets, and the difference from the general military subgroup did not reach statistical significance in the revised exploratory meta-regression. The finding should therefore be regarded as hypothesis-generating rather than as confirmation that intensive voice use independently increases prevalence.

The chemically exposed subgroup had the highest pooled point estimate and differed from the general military subgroup in the exploratory meta-regression. However, this comparison was based on only three studies comprising 145 participants and was markedly imbalanced in sample size relative to the general military subgroup. Differences in population selection, exposure history and outcome ascertainment may have contributed to the observed contrast. Chemical irritants such as sulfur mustard, tear gas and pepper spray can affect the respiratory tract and larynx, potentially producing chronic irritation, inflammation, hypersensitivity and persistent voice symptoms (13–16, 41). The present findings support further investigation of voice and laryngeal outcomes within the broader health assessment of military inhalational exposures, but they do not establish the magnitude or causality of this association.

The review used an intentionally broad umbrella outcome encompassing dysphonia, vocal fatigue, structural vocal-fold pathology, paradoxical vocal fold motion or vocal cord dysfunction, exercise-induced laryngeal obstruction and related laryngeal symptoms. This approach reflected the inconsistent terminology and outcome reporting in the military literature and the shared potential of these conditions to impair voice production, upper-airway function and occupational communication. It did not imply that these outcomes represent a single clinical entity or share the same pathophysiology (41, 42). Combining them within the subgroup-specific syntheses increased the functional and occupational relevance of the review but also contributed to clinical heterogeneity and limits interpretation of each pooled estimate as the prevalence of a specific disorder.

Differences in case ascertainment further affected the estimates. Administrative or ICD-based diagnoses capture clinically documented disorders and may miss mild, unreported or undiagnosed symptoms (17, 23), whereas self-report instruments and screening questionnaires may identify a broader spectrum of functional complaints. In the general military subgroup, the outcome-definition sensitivity analysis produced a substantially lower pooled estimate for ICD-based diagnoses than for self-reported symptoms, while the single clinically assessed study produced a higher estimate. Within the chemically exposed subgroup, clinically assessed dysphonia and questionnaire-based laryngeal hypersensitivity also yielded different estimates, although the number of studies was too small for a reliable formal comparison. These findings indicate that part of the observed heterogeneity was related to what was measured and how cases were identified. Future studies should report clinically diagnosed disorders, structural laryngeal abnormalities, inducible laryngeal obstruction and self-reported voice symptoms separately, even when they are considered collectively in assessments of occupational communication burden.

Active-duty personnel, recruits and veterans were eligible because all may have experienced service-related occupational or environmental hazards, but these populations should not be regarded as directly interchangeable. Active-duty cohorts may underrepresent morbidity because of recruitment standards, medical screening, fitness requirements and retention processes—the “healthy warrior” or “healthy soldier” effect (20, 21, 43). Veterans may differ in age, cumulative exposure, time since exposure, healthcare access and the likelihood of having a clinically documented diagnosis. The small number of available datasets did not permit reliable separate meta-analyses according to service status. In a sensitivity analysis excluding the veteran dataset from the general military subgroup, the pooled estimate changed from 3.3% to 5.7%, but heterogeneity and statistical uncertainty remained extreme. Inclusion of the veteran dataset therefore did not account for the overall heterogeneity, although differences between active-duty and veteran populations remain a potential source of variability. Future studies should report results separately by current service status and duration since military service or relevant exposure.

Civilian occupational voice users provide a relevant, although not directly equivalent, comparison. In a large study of Brazilian teachers, 11.6% of teachers reported a current voice disorder compared with 7.5% of non-teachers, while lifetime voice problems were reported by 63.0% and 35.8%, respectively (19). The 40.0% pooled estimate in the military high-vocal-demand subgroup falls within the broad range reported in civilian voice-intensive occupations, but direct numerical comparison is inappropriate because the military estimate had an extremely wide confidence interval and the included studies differed in outcome definition, population selection and assessment period. Nevertheless, both bodies of evidence support the occupational relevance of sustained voice use, communication in noise and repeated high-intensity phonation (6, 7). The available evidence does not establish whether military personnel in voice-intensive roles have a greater burden than civilian professional voice users.

Given the Very Low certainty of evidence, the present findings do not support a recommendation for universal voice screening or any specific preventive intervention across military populations. Instead, they identify potential priorities for further evaluation within occupational-health and military healthcare systems. Feasibility studies could examine periodic symptom screening in drill instructors, trainers and other communication-intensive roles; voice-health education; assessment of environmental noise and vocal-load conditions; access to amplification or other communication-support systems where operationally appropriate; and structured referral pathways for persistent dysphonia, vocal fatigue or upper-airway symptoms. Following documented chemical or inhalational exposure, it may also be appropriate to investigate whether assessment of laryngeal and voice symptoms adds useful information to established respiratory follow-up.

These proposals were not directly evaluated in the included studies and should be understood as cautious, expert-informed practice considerations rather than evidence-based recommendations. Their clinical utility, acceptability, potential harms, resource implications and effect on occupational functioning would need to be evaluated prospectively before routine implementation.

Future research should prioritize prospective, adequately powered studies using clearly defined and representative military sampling frames. Studies should report numerators and denominators separately by occupational role, current service status, veteran status, sex, age, duration of service and documented vocal, chemical or inhalational exposure. Standardized and validated assessment methods are needed, with separate reporting of self-reported voice symptoms, clinically diagnosed dysphonia, structural vocal-fold pathology, paradoxical vocal fold motion or exercise-induced laryngeal obstruction, and other upper-airway outcomes. Where possible, protocols and analysis plans should be registered before data extraction or analysis. Comparative studies involving military and civilian occupational voice users could help determine whether observed differences are specific to military work or reflect vocal demand more generally. Finally, prospective intervention studies are required before the effectiveness of screening, voice-health education, communication support or early-referral programmes can be established.

### 4.1 Strengths and limitations

This review has several strengths, including PRISMA-based methods, independent study screening and data extraction, a publicly available PROSPERO record, occupationally and environmentally defined subgroups, study-design-specific risk-of-bias assessment, GRADE evaluation and multiple sensitivity analyses. The subgroup-specific approach avoided combining occupationally distinct populations into a single overall prevalence estimate. The statistical approach incorporated restricted maximum likelihood estimation, Hartung–Knapp adjustment, prediction intervals and generalized linear mixed models as a sensitivity analysis.

Several limitations should nevertheless be acknowledged. Heterogeneity was extremely high in the general military and high-vocal-demand subgroups, reflecting substantial differences in study populations, occupational exposures, outcome definitions and the ways in which outcomes were identified or measured. The corresponding confidence and prediction intervals were very wide and, in some analyses, covered almost the entire possible prevalence range. The pooled estimates should therefore be interpreted as descriptive summaries of heterogeneous evidence rather than precise prevalence benchmarks applicable to all military populations. The high-vocal-demand and chemically exposed analyses each contained only three studies, limiting precision and increasing susceptibility to individual-study effects. There was also a marked imbalance in sample size between the general military subgroup and the smaller exposure-defined subgroups, further limiting direct comparisons between their pooled estimates.

The included evidence was observational, generally carried a moderate risk of bias, provided limited adjustment for potential confounders and used inconsistent approaches to voice and laryngeal assessment. Administrative diagnoses, self-reported symptoms, screening questionnaires and clinical assessments may capture different dimensions and levels of morbidity. Although sensitivity analyses according to outcome definition were performed, the small number of studies in each category precluded a definitive explanation of the observed heterogeneity. Formal assessment of small-study effects and publication bias was not considered reliable because each subgroup contained fewer than 10 studies. The exploratory meta-regression was also based on only 11 datasets and had limited statistical power; its subgroup comparisons should therefore not be interpreted as definitive evidence of differences between populations. Overall certainty of evidence was rated as Very Low for all three pooled estimates.

Active-duty personnel, recruits and veterans may differ in exposure history, healthcare access and baseline risk. Although these populations shared service-related occupational or environmental contexts, combining them within broad subgroup categories may have contributed to heterogeneity. The sensitivity analysis excluding the veteran dataset did not resolve the substantial uncertainty. Traumatic brain injury could not be evaluated as a separate subgroup because extractable prevalence data specific to this population were unavailable.

PROSPERO registration was completed after the formal literature searches and study screening, although it preceded data extraction, risk-of-bias assessment and quantitative synthesis. Registration therefore did not provide fully prospective documentation of the search and selection stages. To address this limitation, the timing of registration and all differences between the registered plan and the final analysis have been reported explicitly.

Finally, eligibility was restricted to English-language publications because resources for reliable translation and independent assessment of non-English full texts were unavailable. Consequently, potentially relevant evidence published in other languages may have been missed, and language-selection bias cannot be excluded. Selection effects may also have influenced the estimates in both directions: active-duty cohorts may underestimate morbidity because of military recruitment, retention and fitness requirements, whereas symptomatic or clinically referred cohorts may overestimate disease burden and were therefore not included in the quantitative synthesis.

## 5 Conclusion

The available evidence indicates substantial variation in reported prevalence of voice and related laryngeal disorders across military populations and occupational or environmental risk contexts. Subgroup-specific pooled point estimates were higher in high-vocal-demand and chemically exposed groups than in general military populations; however, extreme heterogeneity, wide confidence and prediction intervals, small numbers of studies and Very Low certainty of evidence preclude interpretation of these estimates as stable prevalence benchmarks or evidence of causal associations.

Voice and laryngeal outcomes may nevertheless represent an under-recognized component of occupational and workforce health in military settings, particularly where service involves intensive voice use or documented chemical or inhalational exposure. Standardized prospective studies are needed to determine the true burden, identify occupationally relevant risk groups and evaluate whether targeted surveillance, prevention and early-referral strategies provide measurable benefit before their routine implementation.

## Data Availability

The original contributions presented in the study are included in the article/Supplementary material, further inquiries can be directed to the corresponding author.
