# Supplementary material for: Prevalence of voice disorders in military personnel: a systematic review and meta-analysis
Source: Front Public Health. 2026 Sep 16;14:1942500. doi: 10.3389/fpubh.2026.1942500 (PMC13623760; doi:10.3389/fpubh.2026.1942500)
Supplement: Supplementary file 1 [file Data_Sheet_2.pdf]

Supplementary File 2

Extended methodological and statistical details and supplementary tables

1. Record management and deduplication

All records retrieved from the searches were imported into EndNote. Duplicate records were identified and removed before screening. The initial search yielded 359 records. After deduplication, 193 unique records remained for title and abstract screening.

2. Study selection and eligibility

Two reviewers independently screened titles and abstracts against the eligibility criteria. Records considered potentially eligible by either reviewer were advanced to full-text assessment. Full texts were then independently assessed by two reviewers. Disagreements were resolved through discussion, and unresolved disagreements were adjudicated by a third reviewer.

Studies were eligible if they included active-duty, reserve, recruit or veteran military populations and reported quantitative data on voice disorders or related laryngeal conditions. Eligible outcomes included dysphonia, vocal fold pathology, paradoxical vocal fold motion/vocal cord dysfunction, exercise-induced laryngeal obstruction, persistent hoarseness, vocal fatigue or other self-reported voice impairment. Studies were required to provide extractable numerators and denominators or sufficient information to calculate prevalence.

Studies were excluded if they were conducted exclusively in civilian populations, were not published in English, did not report original quantitative data, lacked a clear definition of voice disorder or related laryngeal condition, or did not provide extractable prevalence data.

3. Data extraction

Two reviewers independently extracted study characteristics, including author, year, study design, country or military setting, population type, active-duty or veteran status, sample size, number of voice-disorder cases, prevalence estimate, diagnostic or screening method, occupational or environmental risk factors, and eligibility for quantitative synthesis. Extracted exposure categories included high vocal demand, chemical or inhalational exposure, and traumatic brain injury where reported. Disagreements were resolved through discussion or, when necessary, consultation with a third reviewer.

4. Operational definition of voice disorder

For the purposes of this review, voice disorders were defined broadly to reflect the occupational and functional consequences of impaired voice production in military settings. Eligible outcomes included persistent dysphonia, hoarseness, vocal fatigue, aphonia, vocal fold pathology, phonotraumatic lesions, paradoxical vocal fold motion/vocal cord dysfunction, exercise-induced laryngeal obstruction and self-reported voice impairment.

This broad definition was selected because military populations may present with heterogeneous voice-related outcomes depending on the underlying exposure, including high vocal load, noise-related vocal effort, inhalational irritants, chemical exposure, deployment-related respiratory disease or neurological injury. The diagnostic or assessment method used in each study was recorded and considered when interpreting heterogeneity.

5. Definition of military populations

Military personnel were defined broadly to include active-duty service members, recruits, reservists and veterans. Although these groups differ in current occupational exposure and healthcare access, they share service-related risk factors relevant to voice health, including cumulative vocal load, training-related vocal strain, environmental exposures during military service and traumatic injury history.

Where possible, populations were classified according to the dominant exposure or sampling frame:

1. **General military population**: broad military, recruit or veteran cohorts not selected on the basis of high vocal demand, chemical exposure or clinical referral.
2. **High vocal demand**: military personnel in roles requiring sustained or intense voice use, such as drill instructors, military trainers or comparable instructional roles.
3. **Chemically exposed**: cohorts with documented exposure to laryngeal or respiratory irritants, including sulfur mustard, tear gas or pepper spray.
4. **Symptomatic or clinically referred cohorts**: military personnel referred for voice, respiratory or laryngeal symptoms. These cohorts were included in narrative synthesis but excluded from prevalence pooling.

6. Selection of studies for quantitative synthesis

Studies were eligible for quantitative synthesis if they included a defined military-affiliated population, reported a quantitative voice or related laryngeal outcome, provided an extractable numerator and denominator, and could be assigned to one of the prespecified occupational or environmental subgroups. Populations selected exclusively because of voice, respiratory or upper-airway symptoms, or because of referral for specialist assessment, were not included in prevalence pooling.

Study design alone was not a reason for exclusion from the meta-analysis. Cross-sectional, cohort, prevalence and retrospective case-series datasets could be included if they represented a defined military source population and provided an appropriate denominator. Studies of symptomatic or clinically referred patients were retained for narrative synthesis because they provided relevant information on clinical presentation and disease burden, but their inclusion in prevalence pooling would have overestimated prevalence in the underlying military population.

All datasets eligible for quantitative synthesis contained at least one participant with the outcome and at least one participant without the outcome. Therefore, no continuity correction was required, and no dataset was excluded from pooling solely because of a prevalence of 0% or 100%. Studies reporting 100% prevalence were retained in the systematic review but were excluded from quantitative synthesis because they comprised outcome-selected symptomatic or clinically referred cohorts rather than unselected military source populations.

7. Effect measure and transformation

The primary outcome was point prevalence, calculated as the number of participants with the relevant voice or laryngeal outcome divided by the total number of participants in the dataset. Study-specific prevalence estimates and 95% confidence intervals were calculated from the reported numbers of cases and participants.

For the inverse-variance meta-analyses, proportions were transformed to the logit scale:

$$\text{logit}(p) = \ln[p/(1 - p)]$$

The standard error of each logit-transformed proportion was calculated as:

$$SE = \sqrt{[1/e + 1/(n - e)]}$$

where e represents the number of cases and n represents the total sample size. All datasets included in the quantitative synthesis had $e > 0$ and $e < n$. Pooled estimates and their 95% confidence intervals were back-transformed to the proportion scale and reported as percentages.

8. Meta-analysis model

Random-effects meta-analysis was used because true prevalence was expected to vary across military populations, occupational and environmental exposures, study designs and outcome-assessment methods. Logit-transformed proportions were pooled using inverse-variance weighting. Between-study variance (τ^2) was estimated using restricted maximum likelihood (REML).

Confidence intervals for the pooled prevalence estimates were calculated using the Hartung–Knapp adjustment. An ad hoc safeguard was applied when the Hartung–Knapp standard error was smaller than the conventional random-effects standard error. Hartung–Knapp-based 95% prediction intervals, calculated using a t-distribution with $k - 2$ degrees of freedom, were also reported to indicate the range of prevalence that might be expected in a comparable future military population.

Because the included datasets represented clinically and occupationally distinct populations, no overall pooled prevalence estimate was calculated across all studies. Separate random-effects analyses were conducted for:

- general military populations;
- high-vocal-demand personnel;
- chemically exposed populations.

This subgroup-specific approach was used to avoid combining populations with substantially different occupational demands, environmental exposures and expected baseline risks.

9. Heterogeneity assessment

Statistical heterogeneity was quantified using Cochran's Q statistic, the between-study variance (τ^2) and the I^2 statistic. Because I^2 may be unstable or difficult to interpret when only a small number of datasets are available, it was not interpreted using fixed thresholds alone. Its magnitude was considered together with τ^2, the number of datasets, differences in population and outcome definition, and the width of the confidence and prediction intervals.

Forest plots were used to present study-specific prevalence estimates with 95% confidence intervals, subgroup-specific pooled estimates and 95% prediction intervals. When heterogeneity and prediction intervals were very wide, pooled prevalence estimates were interpreted as descriptive summaries of heterogeneous evidence rather than as precise prevalence benchmarks.

10. Subgroup analyses

Subgroup analyses were specified a priori and based on occupational or environmental risk profile. The following subgroups were analysed:

10.1 General military populations

This subgroup included broad military, recruit or veteran cohorts in which participants were not selected for high vocal demand, chemical exposure or clinical referral.

10.2 High-vocal-demand populations

This subgroup included military personnel in roles requiring sustained, intense or forceful voice use. Examples included drill instructors, military trainers and comparable instructional roles. These personnel were expected to have elevated risk because of repeated vocal loading, shouting, command delivery and communication in noisy training environments.

10.3 Chemically exposed populations

This subgroup included cohorts with documented exposure to laryngeal or respiratory irritants, including sulfur mustard, tear gas or pepper spray. These exposures were considered relevant because they may cause acute or chronic laryngeal irritation, inflammation, hypersensitivity or structural injury.

11. Meta-regression and subgroup comparison

Differences between the three predefined subgroups were examined using a mixed-effects meta-regression of logit-transformed prevalence estimates. Between-study variance was estimated using restricted maximum likelihood, and Knapp–Hartung inference was applied to account for uncertainty associated with the small number of datasets. Subgroup membership was entered as a categorical moderator.

The overall moderator effect was evaluated using an omnibus F-test. Pairwise comparisons were obtained by changing the reference category and included:

- high vocal demand versus general military;
- chemical exposure versus general military;
- chemical exposure versus high vocal demand.

Results were reported as differences on the logit scale with 95% confidence intervals and p-values. The proportion of heterogeneity accounted for by subgroup membership (R^2) and residual heterogeneity were also calculated. Because the analysis included only 11 datasets and some subgroups contained only three datasets, the meta-regression was considered exploratory and its results were interpreted cautiously.

12. Sensitivity analyses

Several sensitivity analyses were conducted to examine the robustness of the subgroup-specific prevalence estimates.

First, leave-one-out analyses were performed within each subgroup using the same REML–Hartung–Knapp model as in the primary analysis. Each dataset was sequentially excluded, and the pooled prevalence, 95% confidence interval, τ^2 and I^2 were recalculated. These analyses were interpreted descriptively to identify influential datasets and determine whether exclusion of any single dataset substantially reduced heterogeneity.

Second, subgroup-specific pooled prevalence estimates were recalculated using random-intercept generalised linear mixed models with a binomial likelihood and logit link. These models do not require continuity correction for boundary proportions and were used to assess whether the primary estimates were sensitive to the inverse-variance modelling approach.

Third, sensitivity analyses were conducted according to outcome ascertainment. Datasets were classified as using administrative or ICD-based diagnoses, self-reported symptoms or questionnaires, or clinical or endoscopic assessment. Estimates were pooled only when at least two datasets used a sufficiently comparable method of outcome ascertainment. Categories represented by a single dataset were reported descriptively with an exact binomial 95% confidence interval.

Finally, the general-military analysis was repeated after excluding the respiratory-symptom referral cohort reported by Forbes et al. and, separately, after excluding the veteran cohort reported by Norman et al. These analyses examined the effects of sampling context and service status on the pooled estimate.

Results of the leave-one-out analyses are presented in Supplementary Table S6 and Supplementary Figure S1. Model-comparison, outcome-definition and study-exclusion sensitivity analyses are presented in Supplementary Table S7.

13. Assessment of small-study effects and publication bias

Formal tests of funnel-plot asymmetry and small-study effects were not performed because each subgroup contained fewer than 10 datasets. Under these conditions, statistical tests of asymmetry would have been underpowered and potentially misleading. Publication bias could therefore not be assessed reliably.

14. Risk-of-bias assessment

Risk of bias was assessed independently by two reviewers using tools selected according to study design. Cohort studies were evaluated using the Newcastle–Ottawa Scale (NOS), which assesses selection, comparability and outcome ascertainment. NOS scores of 7–9, 5–6 and 4 or fewer were interpreted as low, moderate and high risk of bias, respectively.

Cross-sectional and prevalence studies were evaluated using the Joanna Briggs Institute (JBI) critical appraisal checklist for studies reporting prevalence data, whereas retrospective case series were evaluated using the JBI checklist for case series. JBI items were rated as Yes, No, Unclear or Not applicable. Overall JBI judgements were based on the number and seriousness of the identified limitations rather than on an unvalidated numerical summary score.

Disagreements between reviewers were resolved through discussion or, when necessary, consultation with a third reviewer. Risk-of-bias assessments informed the interpretation of the results and the GRADE certainty ratings, but no study was excluded solely because of its risk-of-bias rating. Study-level assessments are presented in Supplementary Tables S2–S4.

15. Certainty of evidence

The certainty of evidence for the three subgroup-specific pooled prevalence estimates—general military populations, high-vocal-demand personnel and chemically exposed populations—was assessed independently by two reviewers using the GRADE framework. Five domains were considered:

1. risk of bias;
2. inconsistency;
3. indirectness;
4. imprecision;
5. publication bias.

Because the evidence consisted of observational prevalence studies, certainty began at Low and was downgraded by one or two levels for serious or very serious concerns in the relevant domains. Risk of bias was informed by the NOS and JBI assessments. Inconsistency was evaluated using the magnitude of heterogeneity together with clinical and methodological differences between datasets.
Imprecision was considered serious when the width of the 95% confidence interval exceeded 10 percentage points and very serious when it exceeded 20 percentage points or encompassed substantially different prevalence levels. Publication bias could not be assessed reliably because each subgroup contained fewer than 10 datasets.
Final ratings were classified as High, Moderate, Low or Very low. Disagreements were resolved through discussion or, when necessary, consultation with a third reviewer. Full GRADE evidence profiles are presented in Supplementary Table S5.

16. Statistical software

All revised statistical analyses were conducted in R version 4.6.0. Primary random-effects meta-analyses, prediction intervals, generalised linear mixed models and leave-one-out analyses were performed using the meta package version 8.5-0. Mixed-effects meta-regression was performed using the metafor package version 5.0-1.

.

Supplementary Table S1. Full characteristics of studies included in the systematic review

Study (year)<br>Study type	Population type<br>(Diagnosis/ outcome)	Sample size	Prevalence (%)	SE (%)	95% CI	Included in meta-analysis (reason for exclusion)
Akhavan et al. (2009)<br>Cross-sectional	Chemically exposed<br>(dysphonia)	50	78.0	5.86	64.04 - 88.47	Yes
Balali-Mood et al. (2011)<br>Cross-sectional	Chemically exposed<br>(dysphonia)	43	79.07	6.20	63.96 - 89.96	Yes
Dion et al. (2013)<br>Retrospective cohort	General military<br>(vocal fold paralysis, laryngitis, dysphonia)	1,309,015	0.38	0.01	0.37 - 0.39	Yes
Forbes et al. (2015)<br>Prospective cross-sectional	General military/ post-deployment dyspnoea cohort<br>(dysphonia, vocal fold pathology)	108	22.22	4.00	14.79- 31.24	Yes
Norman et al. (2013)<br>Retrospective case series	General military/ veteran population<br>(organic and functional voice disorders)	303,716	0.35	0.03	0.33 - 0.37	Yes
Perry et al. (2023)<br>Retrospective cohort	General military<br>(dysphonia, vocal nodules, laryngitis)	696	2.73	0.62	1.65 – 4.23	Yes
	High vocal demand<br>(dysphonia, vocal nodules, laryngitis)	3,427	9.07	0.49	8.13 - 10.09	Yes
Sapir et al. (1990)<br>Cross-sectional	High vocal demand/ instructors<br>(hyperfunctional dysphonia)	130	76.92	3.70	68.72- 83.86	Yes
	General military / recruits<br>(abnormal phonation)	386	31.09	2.36	26.50 - 35.97	Yes

Study (year)<br>Study type	Population type<br>(Diagnosis/ outcome)	Sample size	Prevalence (%)	SE<br>(%)	95% CI	Included in meta-analysis (reason for exclusion)
Shah et al. (2022)<br>Cross-sectional	Chemically exposed<br>(Laryngeal hypersensitivity and dysphonia)	52	61.54	6.75	47.02 - 74.70	Yes
Spellman et al. (2020)<br>Cross-sectional	High vocal demand<br>(dysphonia)	151	47.68	4.06	39.50 - 55.96	Yes
Duvall and Dion (2023)<br>Retrospective case series	Symptomatic/ clinically referred<br>(phonotraumatic lesions)	12	100.00	0.00	73.54 – 100.00	No<br>(High-risk symptomatic group)
Gurevich-Uvena et al. (2010)<br>Retrospective case series	Outcome- selected cohort<br>(PVFM/ VCD)	265	100.00	0.00	98.62- 100.00	No<br>(outcome-selected symptomatic cohort)
Morris et al. (1999)<br>Prospective cross-sectional	Symptomatic/ clinically referred<br>(nodules, polyps, or inflammation)	72	13.89	4.08	6.87 – 24.06	No<br>(Symptomatic/ clinically referred cohort)
Nolt et al. (2017)<br>Prospective cross-sectional	Outcome-selected/clinically referred<br>(PVFM / VCD)	100	100.00	0.00	96.38-100.00	No<br>(outcome-selected/clinically referred cohort)
Sapir et al. (1992)<br>Cross-sectional	Reanalysis of survey data<br>(Hyperfunctional dysphonia and related disorders)	443	54.18	2.37	49.41 - 58.89	No<br>(reanalysis of previously reported data; not an independent dataset)
Morris et al. (2002)<br>Retrospective cohort	Symptomatic/clinically referred<br>(benign lesions or chronic laryngitis)	105	9.52	2.86	4.66 – 16.82	No<br>(symptomatic/clinically referred cohort)
Morris et al. (2013)<br>Retrospective cohort	Outcome-selected/clinically referred<br>(VCD or persistent laryngeal inflammation)	48	100.00	0.00	92.60- 100	No<br>(outcome-selected/clinically referred cohort)

CI, confidence interval; PVFM, paradoxical vocal fold motion; SE, standard error; VCD, vocal cord dysfunction. Study-specific confidence intervals were calculated using the exact binomial method. Perry et al. (2023) and Sapir et al. (1990) each contributed two non-overlapping datasets representing different military subgroups. Studies reporting 100% prevalence were excluded from quantitative synthesis because they comprised outcome-selected symptomatic or clinically referred cohorts, not because of the boundary prevalence value itself.

Supplementary Table S2. Risk of bias assessment using the Newcastle–Ottawa Scale (NOS)

Study (Year)	NOS Score	Risk of Bias
Dion et al. (2013)	6	Moderate
Morris et al. (2002)	5	Moderate
Morris et al. (2013)	6	Moderate
Perry et al. (2023)	6	Moderate

NOS, Newcastle-Ottawa Scale. Scores of 7–9, 5–6 and 4 or fewer were interpreted as low, moderate and high risk of bias, respectively. Perry et al. (2023) contributed two non-overlapping datasets to the meta-analysis, but risk of bias was assessed once at the study level.

Common limitations included limited adjustment for potential confounding, reliance on routinely collected or retrospective data, non-random sampling or clinical referral, and variation in outcome ascertainment.

Supplementary Table S3. Risk-of-bias assessment using the Joanna Briggs Institute checklist for prevalence studies

Study (Year)	Q1	Q2	Q3	Q4	Q5	Q6	Q7	Q8	Q9	Overall Risk of Bias
Akhavan et al. (2009)	Y	Y	N	Y	Y	Y	Y	Y	U	Moderate
Balali-Mood et al. (2011)	Y	U	N	Y	Y	Y	Y	Y	U	Moderate
Forbes et al. (2015)	N	N	N	Y	Y	Y	Y	Y	U	High
Morris et al. (1999)	U	U	N	Y	Y	Y	Y	Y	NA	Moderate
Nolt et al. (2017)	U	U	N	Y	Y	Y	Y	Y	NA	Moderate
Sapir et al. (1990)	Y	U	Y	Y	Y	Y	Y	Y	U	Moderate
Sapir et al. (1992)	Y	U	Y	Y	Y	Y	Y	Y	U	Moderate
Shah et al. (2022)	Y	U	N	Y	Y	Y	Y	Y	U	Moderate
Spellman et al. (2020)	Y	Y	Y	Y	Y	Y	Y	Y	U	Moderate

JBI, Joanna Briggs Institute; Y, Yes; N, No; U, Unclear; NA, Not applicable. Q1, appropriate sampling frame; Q2, appropriate sampling; Q3, adequate sample size; Q4, detailed description of participants and setting; Q5, sufficient data coverage; Q6, valid identification of the condition; Q7, standard and reliable measurement; Q8, appropriate statistical analysis; Q9, adequate response rate or appropriate management of non-response. Overall ratings were based on the number and seriousness of identified limitations rather than on an unvalidated numerical summary score. The table presents a condensed domain-level summary derived from the JBI critical appraisal checklist for studies reporting prevalence data.

Supplementary Table S4. Risk-of-bias assessment using the Joanna Briggs Institute checklist for case series

Study	Q1	Q2	Q3	Q4	Q5	Q6	Q7	Q8	Q9	Q10	Overall Risk of Bias
Duvall and Dion (2023)	Y	Y	Y	U	U	Y	Y	Y	Y	Y	Moderate
Gurevich-Uvena et al. (2010)	Y	Y	Y	U	U	Y	Y	Y	Y	Y	Moderate
Norman et al. (2013)	Y	Y	Y	Y	Y	Y	Y	Y	Y	Y	Low

JBI, Joanna Briggs Institute; Y, Yes; N, No; U, Unclear; NA, Not applicable. Q1, clear inclusion criteria; Q2, condition measured in a standard and reliable way; Q3, valid methods for identifying the condition; Q4, consecutive inclusion; Q5, complete inclusion; Q6, clear participant demographics; Q7, clear clinical information; Q8, clear reporting of outcomes or follow-up; Q9, clear site information; Q10, appropriate statistical analysis. Overall ratings were based on the number and seriousness of identified limitations rather than on an unvalidated numerical summary score.

Across all 16 included studies, one study was rated as having low risk of bias, 14 as having moderate risk of bias and one as having high risk of bias.

Supplementary Table S5. GRADE certainty of evidence ratings

Outcome	Risk of Bias	Inconsistency	Indirectness	Imprecision	Publication Bias	Overall Certainty
**General military population**	Serious (–1)	Very erious (–2)	Not Serious (0)	Very Serious (–2)	Not assessed reliably *	**Very low**
**High-vocal-demand personnel**	Serious (–1)	Very Serious (–2)	Not Serious (0)	Very Serious (–2)	Not assessed reliably *	**Very low**
**Chemically exposed cohorts**	Serious (–1)	Serious (–1)	Serious (–1)	Very Serious (–2)	Not assessed reliably *	**Very low**

Observational prevalence evidence started at Low certainty. Ratings were downgraded for serious or very serious concerns but could not be rated below Very low.

*Publication bias could not be assessed reliably because each subgroup contained fewer than 10 datasets.

Supplementary Table S6. Leave-one-out sensitivity analyses

Subgroup	Excluded dataset	Pooled Prevalence (%)	95% CI (%)	τ^2	I^2 (%)
**General military population**	None	3.30	0.19–37.54	5.3114	99.8
	Dion et al. (2013)	5.61	0.16–68.30	5.0615	99.9
	Norman et al. (2013)	5.71	0.18–67.60	4.9131	99.9
	Forbes et al. (2015)	1.98	0.05–43.50	5.2198	99.8
	Perry et al. (2023) - general	3.47	0.05–71.20	7.0612	99.9
	Sapir et al. (1990) - recruits	1.75	0.07–32.80	4.2875	99.3
**High-vocal-demand personnel**	None	40.0	0.80–98.22	3.1315	99.5
	Perry et al. (2023) – high vocal demand	63.39	0.05–100.00	0.8058	95.8
	Sapir et al. (1990) - instructors	23.08	0.00–100.00	2.4308	99.4
	Spellman et al. (2020)	36.45	0.00–100.00	6.1313	99.6
**Chemically Exposed cohorts**	None	72.81	43.30–90.38	0.1448	57.5
	Akhavan et al. (2009)	70.37	1.02–99.82	0.2582	70.0
	Balali-Mood et al. (2011)	69.97	1.48–99.72	0.2176	68.8
	Shah et al. (2022)	78.49	12.87–98.90	0.0000	0.0

CI, confidence interval. "None" denotes the primary analysis with no dataset excluded. Each subsequent row presents the pooled estimate after exclusion of the indicated dataset. Estimates were calculated using random-effects models of logit-transformed proportions with restricted maximum likelihood estimation and Hartung–Knapp adjustment. τ^2 is reported on the logit scale.

Supplementary Table S7. Model-comparison, outcome-definition and study-exclusion sensitivity analyses

Panel A. Comparison of the primary REML–Hartung–Knapp model with the GLMM sensitivity model

Subgroup	Model	Datasets (k)	Cases/participants	Pooled prevalence, % (95% CI)	95% prediction interval (%)	I^2 (%)
General military	REML–Hartung–Knapp	5	6,139/1,613,921	3.30 (0.19–37.54)	0.001–99.06	99.8
	GLMM	5	6,139/1,613,921	3.27 (0.55–17.01)	—	99.8
High vocal demand	REML–Hartung–Knapp	3	483/3,708	40.00 (0.80–98.22)	0.00–100.00	99.5
	GLMM	3	483/3,708	39.99 (11.37–77.59)	—	99.5
Chemical exposure	REML–Hartung–Knapp	3	105/145	72.81 (43.30–90.38)	0.64–99.91	57.5
	GLMM	3	105/145	72.88 (62.60–81.18)	—	57.5

Panel B. Sensitivity analyses according to outcome assessment method

Subgroup	Outcome assessment method	Synthesis	Datasets (k)	Cases/participants	Prevalence, % (95% CI)	I^2 (%)
General military	Administrative or ICD-based diagnosis	Pooled	2	5,976/1,612,731	0.36 (0.22–0.60)	81.6
	Self-reported symptoms	Pooled	2	139/1,082	10.18 (0.00–100.00)	99.1
	Clinical or endoscopic assessment	Single dataset	1	24/108	22.22 (14.79–31.24)	—
High vocal demand	Self-reported symptoms or questionnaires	Pooled	3	483/3,708	40.00 (0.80–98.22)	99.5
Chemical exposure	Clinical dysphonia assessment	Pooled	2	73/93	78.49 (12.87–98.90)	0.0
	LHQ screening questionnaire	Single dataset	1	32/52	61.54 (47.02–74.70)	—

Panel C. Study-exclusion sensitivity analyses in the general military subgroup

Analysis	Datasets (k)	Cases/participants	Pooled prevalence, % (95% CI)	95% prediction interval (%)	I^2 (%)
**Primary analysis**	5	6,139/1,613,921	3.30 (0.19–37.54)	0.001–99.06	99.8
**Excluding Forbes et al. (2015)**	4	6,115/1,613,813	1.98 (0.05–43.50)	<0.01–99.92	99.8
**Excluding Norman et al. (2013)**	4	5,084/1,310,205	5.71 (0.18–67.60)	<0.01–99.96	99.9

CI, confidence interval; GLMM, generalised linear mixed model; ICD, International Classification of Diseases; LHQ, Laryngeal Hypersensitivity Questionnaire; PI, prediction interval; REML, restricted maximum likelihood. Dashes indicate that a prediction interval was not calculated for the GLMM sensitivity model or that I^2 was not applicable to a single-dataset estimate. Pooled estimates were calculated using REML with Hartung–Knapp adjustment unless otherwise specified. Confidence intervals for single-dataset estimates were calculated using the exact binomial method.

Supplementary Figures

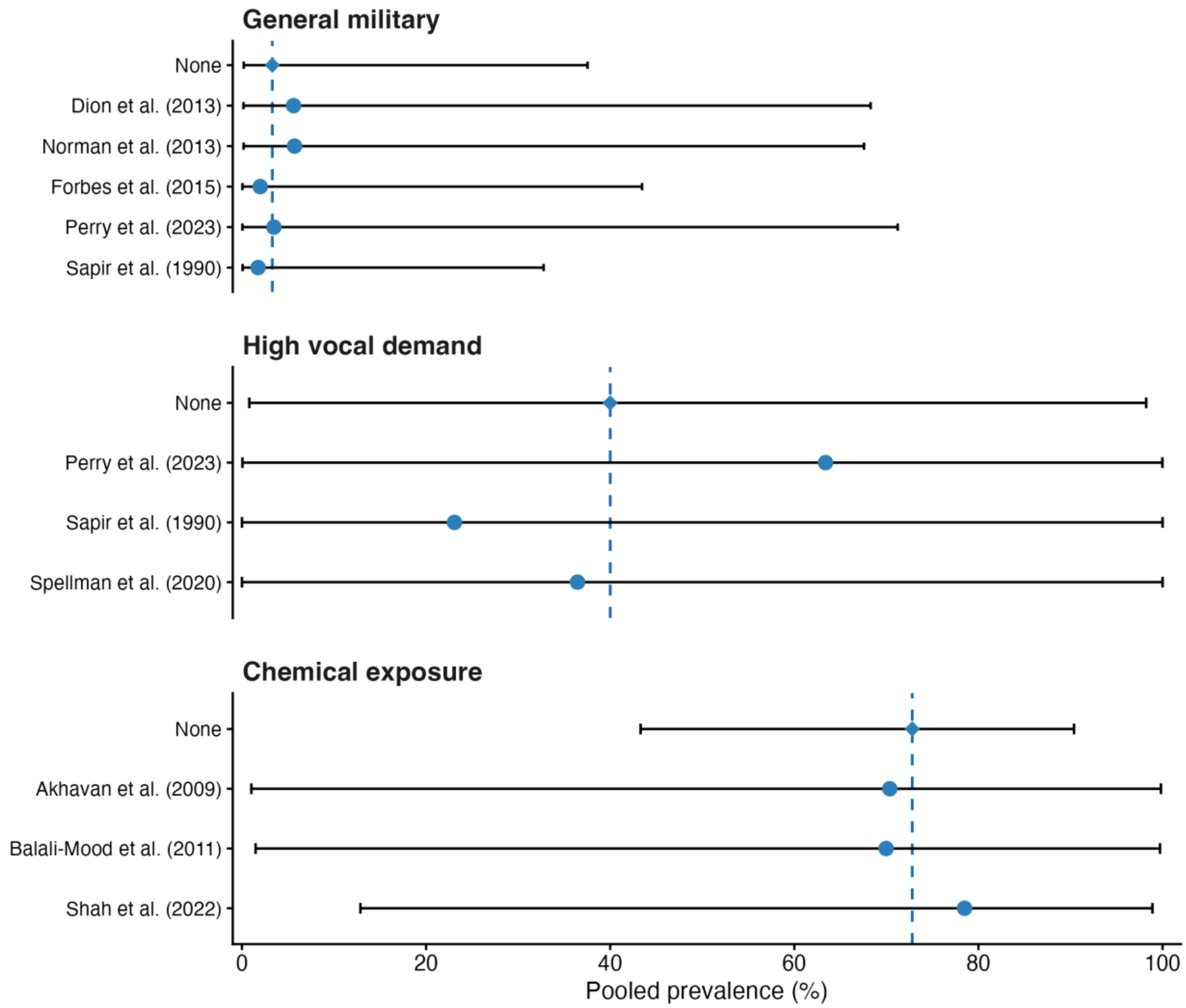

Supplementary Figure S1. Leave-one-out sensitivity analyses by military subgroup.
Points represent pooled prevalence estimates with 95% confidence intervals obtained after sequential exclusion of each dataset. “None” denotes the primary analysis with no dataset excluded, and dashed vertical lines indicate the primary subgroup estimates. CI, confidence interval.
