# Supplementary material for: Prevalence of voice disorders in military personnel: a systematic review and meta-analysis
Source: Front Public Health. 2026 Sep 16;14:1942500. doi: 10.3389/fpubh.2026.1942500 (PMC13623760; doi:10.3389/fpubh.2026.1942500)
Supplement: Supplementary file 2 [file Data_Sheet_3.pdf]

PRISMA 2020 Checklist – Prevalence of Voice Disorders in Military Personnel: A Systematic Review and Meta-Analysis

Item	Location where reported
1. Title	Title, main manuscript, p. 1.
2. Abstract	Structured Abstract, main manuscript, pp. 1.
3. Rationale	Introduction, main manuscript, pp. 2–3.
4. Objectives	Introduction, final paragraph, main manuscript, p. 3.
5. Eligibility criteria	Section 2.2, Eligibility criteria, main manuscript, p. 4.
6. Information sources	Section 2.3, Search strategy and study selection, main manuscript, p. 5; Supplementary File 1.
7. Search strategy	Section 2.3, Search strategy and study selection, main manuscript, p. 4; complete database-specific search strategies and search dates are provided in Supplementary File 1.
8. Selection process	Sections 2.1 and 2.3, main manuscript, pp. 3 and 5.
9. Data collection process	Section 2.4, Data extraction, main manuscript, p. 5.
10a. Outcomes	Section 2.6, Outcomes and subgroup definitions, main manuscript, pp. 6-7
10b. Other variables	Sections 2.4 and 2.6, main manuscript, pp. 5–7.
11. Risk of bias assessment	Section 2.5, Risk of bias and certainty of evidence, main manuscript, pp. 5–6; Supplementary Tables S2–S4, Supplementary File 2, pp. 11–12.
12. Effect measures	Sections 2.6 and 2.7, main manuscript, pp. 6-7
13a. Eligibility for each synthesis	Sections 2.2 and 2.6, main manuscript, pp. 4 6 -7
13b. Data preparation	Section 2.7, Statistical analysis, main manuscript, p. 7.
13c. Methods for tabulation/display	Section 2.7, main manuscript, p. 7 Tables 1–3; Figures 1-2; Supplementary Tables S1–S7 and Supplementary Figure S1..

Item	Location where reported
13d. Synthesis methods	Section 2.7, Statistical analysis, main manuscript, p 7.
13e. Exploration of heterogeneity	Section 2.7, main manuscript, p. 7; Section 3.4, main manuscript, p. 10; Table 3, p. 22
13f. Sensitivity analyses	Section 2.7, main manuscript, p. 7; Section 3.5, main manuscript, pp. 10–11; Supplementary Tables S6- S7 and Supplementary File 2, pp.13-15, Supplementary Figure S, p. 16..
14. Reporting bias assessment	Section 2.7, main manuscript, p. 7; Assessment of small-study effects and publication bias, Supplementary File 2, p. 7. Formal tests were not performed because each subgroup contained fewer than 10 datasets.
15. Certainty assessment	Section 2.5, main manuscript, pp. 4–5; Supplementary Table S5, Supplementary File 2, pp. 12–13.
16a. Study selection	Section 3.1, Study selection and study characteristics, main manuscript, p. 8; Figure 1.
16b. Excluded studies	Figure 1, aggregate reasons for exclusion following full-text assessment.
17. Study characteristics	Section 3.1, main manuscript, p. 8; Table 1, p. 20; Supplementary Table S1, Supplementary File 2, pp. 9–10.
18. Risk of bias in studies	Section 3.2, main manuscript, p. 9; Supplementary Tables S2–S4, Supplementary File 2, pp. 11–12.
19. Results of individual studies	Section 3.3, main manuscript, pp. 9-10; Figure 2; Supplementary Table S1, Supplementary File 2, pp. 9–10.
20a. Results of syntheses – contributing studies	Section 3.1, main manuscript, p. 8; Table 1, p. 20; Supplementary Table S1, Supplementary File 2, pp. 9–10.
20b. Results of statistical syntheses	Section 3.3, main manuscript, pp. 9–10; Table 2, p. 21; Figure 2.
20c. Investigations of heterogeneity	Section 3.4, main manuscript, p. 10; Table 3, p. 22.
20d. Sensitivity analyses	Section 3.5, main manuscript, pp. 10–11; Supplementary Table S6, Supplementary File 2, p. 13; Supplementary Table S7, pp. 14–15; Supplementary Figure S1, p. 16.
21. Reporting	Section 3.5, main manuscript, p. 11. Publication bias could not be assessed

Item	Location where reported
biases	reliably because each subgroup contained fewer than 10 datasets.
22. Certainty of evidence	Section 3.2, main manuscript, p. 9; Supplementary Table S5, Supplementary File 2, pp. 12–13.
23a. Interpretation of results	Discussion, main manuscript, pp. 12–14.
23b. Limitations of included evidence	Strengths and limitations, main manuscript, pp. 14–15.
23c. Limitations of review process	Strengths and limitations, main manuscript, pp. 14–15.
23d. Implications for practice, policy and research	Discussion, main manuscript, pp. 13–14; Conclusion, p. 15.
24a. Registration	Section 2.1, Protocol and registration, main manuscript, p. 3; PROSPERO CRD420250640753.
24b. Protocol access	Section 2.1, main manuscript, p. 3; the public PROSPERO record is available at https://www.crd.york.ac.uk/PROSPERO/view/CRD420250640753; no separate full protocol document was prepared.
24c. Amendments to protocol	Section 2.1, main manuscript, p. 3; deviations from the registered plan and their rationale are reported.
25. Support	Funding statement, main manuscript, p. 15.
26. Competing interests	Conflict of interest statement, main manuscript, p. 15.
27. Availability of data, code and materials	Data availability statement, main manuscript, p. 15; Supplementary Files 1 and 2.
